# Potential Antiosteoporotic Agents from Plants: A Comprehensive Review

**DOI:** 10.1155/2012/364604

**Published:** 2012-12-31

**Authors:** Min Jia, Yan Nie, Da-Peng Cao, Yun-Yun Xue, Jie-Si Wang, Lu Zhao, Khalid Rahman, Qiao-Yan Zhang, Lu-Ping Qin

**Affiliations:** ^1^Department of Pharmacognosy, School of Pharmacy, Second Military Medical University, Shanghai 200433, China; ^2^Department of Pharmacy, Fujian University of Traditional Chinese Medicine, Fuzhou 350108, China; ^3^School of Pharmacy and Biomolecular Sciences, Liverpool John Moores University, Byrom Street, Liverpool L3 3AF, UK

## Abstract

Osteoporosis is a major health hazard and is a disease of old age; it is a silent epidemic affecting more than 200 million people worldwide in recent years. Based on a large number of chemical and pharmacological research many plants and their compounds have been shown to possess antiosteoporosis activity. This paper reviews the medicinal plants displaying antiosteoporosis properties including their origin, active constituents, and pharmacological data. The plants reported here are the ones which are commonly used in traditional medical systems and have demonstrated clinical effectiveness against osteoporosis. Although many plants have the potential to prevent and treat osteoporosis, so far, only a fraction of these plants have been thoroughly investigated for their physiological and pharmacological properties including their mechanism of action. An attempt should be made to highlight plant species with possible antiosteoporosis properties and they should be investigated further to help with future drug development for treating this disease.

## 1. Introduction

Osteoporosis is a systemic skeletal disease characterized by low bone mineral density (BMD) and microarchitectural deterioration of bone tissue, leading to a consequent increase in bone fragility and fracture risk. Hypogonadism is the most well-established cause of osteoporosis, which is usually thought to be an age-adjusted symptom [1]. In recent years, it has become a major health hazard afflicting more than 200 million people worldwide and has one of the highest incidence of all diseases in the elderly population [2]. The Health Departments in many countries are spending large amounts of money investigating new antiosteoporosis drugs every year. 

Based on the principles of physiological bone regeneration and the role of osteoblasts and osteoclasts in the process, it is obvious that the rate of supply of new osteoblasts and osteoclasts, and the timing of the death of these cells by apoptosis are critical determinants of bone regeneration [3]. The activities of these cells are mainly associated with sex steroid deficiency, senescence, and glucocorticoid excess; furthermore, at menopause, the rate of bone remodeling increases precipitously. The loss of sex steroids upregulates the formation of osteoclasts and osteoblasts in the marrow by upregulating the production and action of cytokines, including IL-6, TNF, IL-1, and macrophage colony stimulating factors (M-CSF) which mediate osteoclastogenesis and osteoblastogenesis [4]. The imbalances between bone resorption and formation are due to an extension of the working lifespan of the osteoclasts and shortening of the working lifespan of the osteoblasts. The amount of bone formed during each remodeling cycle decreases with age in both sexes. In aging women, even in extreme old age, bone turnover is most likely increased by secondary hyperparathyroidism or by the continuing effect of estrogen deficiency [5]. Glucocorticoid excess decreases intestinal calcium absorption and hypercalciuria due to defective vitamin D metabolism. These changes result in increased bone resorption, decreased osteoblast proliferation and biosynthetic activity, and sex-steroid deficiency, as well as hyperparathyroidism [6]. Glucocorticoid excess has a suppressive effect on osteoblastogenesis in the bone marrow and also promotes the apoptosis of osteoblasts and osteocytes. Glucocorticoids directly suppress BMP-2 (bone morphogenetic protein-2) and Cbf*α*-1 (core binding factor*α*1), two critical factors for osteoblastogenesis, and may also decrease the production of IGFs (insulin-like growth factors) while stimulate the transcriptional activity of PPAR-*γ*2 (peroxisome proliferator-activated receptor-*γ*2) [7].

There are certain risk factors which differ among individuals and are linked to the development of osteoporosis and contribute to the likelihood of developing the disease. These factors can be divided into two categories, the first being non-modifiable factors such as gender, age, body size, ethnicity, and family history, the other modifiable factors are sex hormones, anorexia nervosa, calcium and vitamin D intake, medication use, lifestyle, cigarette smoking, and alcohol intake, and so forth [2]. Physical exercise, dietary supplement, and pharmacotherapy are usually used for prevention and treatment of osteoporosis. The pharmacotherapy for osteoporosis is usually focused on accommodating the estrogen level or bone remodel. The mechanisms involves many aspects, such as stimulating parathyroid hormone (PTH) synthesizes; inducing the expression of OPG (osteoprotegerin); decreasing IL-1, 4, 6, and M-CSF; increasing estrogens or like-estrogens; supplementing Ca, P in bones; to inhibit the proliferation of osteoclast and induce osteoclast apoptosis; and to enhance the proliferation and differentiation of osteoblast. The drugs used mainly include estrogen, parathyroid hormone (PTH), various bisphosphonates, the selective oestrogen-receptor modulators (SERM) raloxifene, calcitonin, sodium fluoride, and calcium and vitamin D [8].

Calcium supplementation alone provides small beneficial effects on bone mineral density through postmenopausal life and may slightly reduce fracture rates, and vitamin D may be effective in deficient individuals [9]. The long-term hormone therapy for osteoporosis of postmenopausal women is controversial, because of increases in the risk of breast carcinoma, endometrial cancer, and cardiovascular disease. In postmenopausal women with osteoporosis and cardiovascular risk factors, combined oestrogen and progestagen or estrogen alone therapy should be avoided in favor of alternative antiresorptive agents. Hormone therapy remains an option only for short-term early use around the menopause in symptomatic women with high rates of risk fracture [10]. Bisphosphonates can reduce the risk of vertebral fractures and non-vertebral fractures including hip fractures. The dosing regimen (which require the patients to fast and remain upright for at least 30 min) and upper gastrointestinal side effects are often limiting factors in daily bisphosphonate therapy. Their duration of physiological effect is unclear, but bone turnover makers can remain suppressed for at least 5 years after their discontinuation [11]. Selective oestrogen-receptor modulators (including raloxifene, arzoxifene and lasofoxifene) are a chemically diverse set of compounds that do not have the steroid structure of oestrogen, but have a tertiary structure that allows binding to the oestrogen receptor to exert selective agonist or antagonist effects on different oestrogen target tissue. The most studied is raloxifene; its effects on markers of bone turnover and bone mineral density have generally been less than with biophosphonate therapy, so it should probably mainly be used in postmenopausal women with milder osteoporosis or in those with predominantly spinal osteoporosis. Potential side effects include an increase risk of venous thrombosis similar to that with hormone therapy and exacerbation of hot flushes [12]. The previously mentioned therapies act mainly to reduce bone resorption and the anabolic agent parathyroid hormone (PTH) mainly stimulates bone formation. The clinical trials in postmenopausal women showed PTH reduced the risk of fractures with 20 *μ*g dose. However, the benefit in terms of bone mineral density seemed to wane after discontinuation unless followed by an antiresorptive agent [13]. In addition, strontium ranelate is a fairly new antiosteoporotic agent that has been approved in the European Union for the treatment of postmenopausal osteoporosis. It increase bone formation while reducing bone resorption, however its mechanism of action remains unclear. The clinical trials in postmenopausal women show that strontium ranelate reduces the risk of fractures and was well tolerated apart from a low rate of gastrointestinal side- effects and an increased risk of venous thrombosis [14].

Estrogen, bisphosphonates, calcitonin, calcium products, ipriflavone, and anabolic steroids are clinically used as effective medications [15]; however, each of them has established some side effects. Many medicinal plants have long been used to prevent and treat osteoporosis in many countries. These natural medicines derived from plants have fewer side effects and are more suitable for long-term use than synthesized drugs. These plant medicines containing numerous chemical constituents usually exert their therapeutic effects through multipathways and have multitargets, this property is parallel with the multiple factors of osteoporosis pathogenesis. In this paper, we summarize recent studies about antiosteoporotic medicinal plants with particular emphasis on the chemical constituents, mechanisms of action, and therapeutic applications. This will provide more information for the applications of medicinal plants in the prevention and treatment of osteoporosis.

## 2. Materials and Methods

The following computerized databases were searched from their inception to May 2012: MEDLINE (PUBMED), ALT HEALTH WATCH (EBSCO), and Google scholar. Text word search of titles and abstracts was conducted using the following entries in various conjunction or disjunction: osteoporosis, osteoblast, osteoclast, herbs, medicinal plant, natural product, herbal medicine, plant medicine, and phytomedicine. Each study included in this paper satisfies the following criteria: (i) the studies on antiosteoporotic activity were conducted on animal, or cultured osteoblast and osteoclast, and (ii) plant extracts, or compounds isolated from plant. The exclusion criteria consisted of (i) the herb studied was an herbal formula (i.e., neither a single herb nor a single herbal compound), (ii) the articles were not written in English or translated into English. Two reviewers independently extracted the data and performed quality assessment.

## 3. Results

### 3.1. Medicinal Herbs

As shown in Table 1, literature survey showed that 76 medicinal plants were reported in ethnopharmacological studies for their potential benefits in osteoporosis treatment. These plants were distributed among 44 families, including Amaranthaceae (1 spp), Amaryllidaceae (1 spp), Apiaceae (6 spp), Berberidaceae (5 spp), Brassicaceae (1 spp), Campanulaceae (1 spp), Caprifoliaceae (1 spp), Compositae (4 spp), Convolvulaceae (1 spp), Davalliaceae (1 spp), Dicksoniaceae (1 spp), Dioscoreaceae (2 spp), Dipsacaceae (1 spp), Ericaceae (1 spp), Eucommiaceae (1 spp), Euphorbiaceae (1 spp), Fabaceae (11 spp), Ginkgoaceae (1 spp), Juglandaceae (1 spp), Labiatae (2 spp), Lauraceae (1 spp), Liliaceae (5 spp), Lythraceae (1 spp), Malvaceae (1 spp), Menispermaceae (1 spp), Myrsinaceae (1 spp), Oleaceae (1 spp), Orchidaceae (1 spp), Orobanchaceae (2 spp), Pleurotaceae (1 spp), Polypodiaceae (1 spp), Punicaceae (1 spp), Ranunculaceae (2 spp), Rosaceae (2 spp), Rubiaceae (1 spp), Rutaceae (2 spp), Scrophulariaceae (1 spp), Solanaceae (1 spp), Taxaceae (1 spp), Theaceae (1 spp), Ulmaceae (2 spp), Verbenaceae (1 spp), Vitaceae (1 spp), and Zingiberaceae (1 spp). The evaluations of antiosteoporotic activity of these plants are based on the animal experiment (58 spp), cultured osteoblast, and osteoclast in vitro (18 spp). The more highly represented botanic families were: Fabaceae (11 spp), Apiaceae (6 spp), Liliaceae (5 spp), and Compositae (4 spp). Among plant parts, root and rhizome (28 spp) were maximally utilized for antiosteoporosis. Among various parts of plants used in bone metabolism regulation, are root and rhizome (28 spp), fruit and seed (21 spp), stem and bark (13 spp), leaf (7 spp), whole plant and aerial parts (6 spp), and flower (1 spp). Multiple references were consulted for detailed information on research status of 10 plant species which are discussed below.

#### 3.1.1. Epimedium Plants

**Figure 1:**
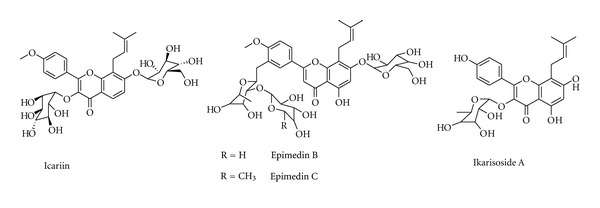
Chemical structure of compounds from *Epimedium* plants.

*Epimedium* (Berberidaceae) is a low-growing, deciduous, perennial plant. The leaves of *E. brevicornum *Maxim., *E. sagittatum *(Sieb.et Zucc.) Maxim., *E. pubescens* Maxim., *E. wushanense *T. S. Ying, and *E. koreanum* Nakai have long been used to prevent and treat osteoporosis and other menopause diseases in China. These are the most frequently used herb drugs in antiosteoporotic Chinese traditional medicine formula [16]. Flavonoids including icariin, epimedin B, and epimedin C (Figure 1) are the main antiosteoporotic constituents, which inhibit bone resorption, stimulate bone formation, suppress urinary calcium excretion, and accordingly prevent osteoporosis without hyperplastic effects on the uterus in the ovariectomized (OVX) rat model [17]. The flavonoids from *Epimedium* plants possess an estrogen-like activity and modulate the bone metabolism through estrogen receptor pathway, and may improve the development of osteoblasts by promoting the ALP (alkaline phosphatase) activity through regulating the expression of IL-6, OPG, RANKL (receptor activator of nuclear factor-*κ*B ligand), M-CSF, Cbf*α*1 (core binding factor*α*1), BMP-2 and SMAD4 involved in the bone remodel and modulate proliferation and activity of osteoblasts and osteoclasts [18, 19]. *Epimedium *flavonoids enhance the mRNA expression of BMP-2, BMP-4, Runx2 (Runt-related transcription factor 2), and cyclinD1, all of which are BMP or Wnt-signaling pathway related regulators, indicating that *Epimedium *flavonoids exerts promoting effects on osteogenic differentiation, which plausibly functions via the BMP and Wnt/*β*-catenin signaling pathways [20].

Icariin (Figure 1), the main active flavonoid glucoside isolated from *Epimedium* plant, is found to have a therapeutic effect on osteoporosis in ovariectomy rat models and postmenopausal women and has been shown to suppress the loss of bone mass and strength in distal femur in tibia following OVX through increasing the mRNA expression ratio of OPG/RANKL [21, 22]. Icariin increases estrogen receptor (ER) dependent cell proliferation, ALP activity, and the OPG/RANKL ratio in UMR 106 cells, and increases ER*α* phosphorylation, showing that icariin exerts anabolic effects in bone possibly by activating ER [23]. In addition, icariin decreases the TRAP activity of osteoclasts, reduces the size of LPS-induced osteoclasts formation without inhibition of cell viability, inhibits LPS-induced bone resorption and the expression of IL-6 and TNF-*α*. The synthesis of cyclooxygenase type-2 (COX-2) and prostaglandin E_2_ (PGE_2_), and expression of LPS-induced hypoxia inducible factor-1*α* (HIF-1*α*) in osteoclasts, LPS-mediated activation of the p38 and JNK on osteoclasts is also inhibited. It also reduces the LPS-induced activation of ERK1/2 and I*κ*-B*α*, indicating that icariin has an in vitro inhibitory effect on osteoclasts differentiation that can prevent inflammatory bone loss by suppressing activation of the p38 and JNK pathway [24].

Ikarisoside A (Figure 1), a natural flavonoid isolated from *E. koreanum, *exerts antioxidant potential and anti-inflammatory effects in LPS-stimulated bone marrow-derived macrophage precursor cells and RAW 264.7 cells, also inhibits osteoclastogenesis in RANKL-stimulated RAW 264.7 cells as well as in bone marrow-derived macrophages [25]. Ikarisoside A has been found to decrease the osteoclast-specific genes, like matrix metalloproteinase 9 (MMP-9), tartrate-resistant acid phosphatase (TRAP), receptor activator of NF-*κ*B (RANK), and cathepsin K, and blocks the resorbing capacity of RAW 264.7 cells on calcium phosphate-coated plates, and inhibits the RANKL-mediated activation of NF-*κ*B, JNK, and Akt. This indicates that Ikarisoside A has potential for use in treatment of diseases involving abnormal bone lysis such as osteoporosis, rheumatoid arthritis, and periodontal bone erosion [26].

#### 3.1.2. *Glycine max*  L.

**Figure 2:**
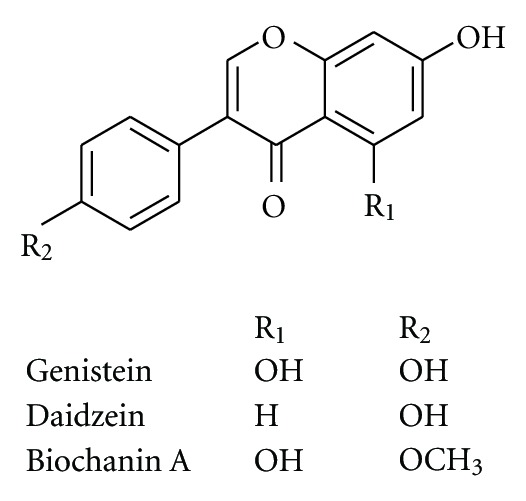
Chemical structure of compounds from *Glycine max *L.

*Glycine max *L. (Fabaceae) originally grows in the southwest of Asia, and is now widely planted in warm areas. Its seed, also called soybeanis a common dietary supplement, and contains plenty of nutritional substances, such as proteins and flavonoids including genistein, daidzein and biochanin A (Figure 2). The soy flavonoids which are structurally and functionally related to 17-beta-estradiol have strong effects on bone metabolism in postmenopausal women and have a role in the prevention and treatment of postmenopausal osteoporosis [27]. Epidemiological studies and clinical trials suggest that soy isoflavones have beneficial effects on bone mineral density, bone turnover markers, and bone mechanical strength in postmenopausal women. The diet containing 22% soybean protein can be just as effective as daily estrogen administration in suppressing bone loss induced by ovariectomy. However, unlike estrogen, a soybean protein diet does not have uterotrophic side effects, and does not decrease the markers of bone turnover. The modulation of soybean protein and flavonoids on nuclear receptors focuses especially on the expression of receptors for estrogens, progesterone, androgen, vitamin D, retinoic acid, and thyroid hormones as well as the potential impact on physiological functions [28]. Soy flavonoids can modulate trabecular microstructural properties, inhibit bone loss in both osteoporotic animal models and postmenopausal women by regulating bone metabolism-related gene expression, including calciotropic receptor, cytokines, growth factors, ALP, collagen type I (COL I), and osteocalcin. In addition, soy flavonoids, phytoestrogen, in chemical structure, are antiestrogenic on both ER alpha and ER beta-dependent gene expression in the brain and estrogen-dependent behavior [29, 30]. 

Genistein (Figure 2) exhibits estrogenic action in bone and bone marrow to regulate B-lymphopoiesis and prevent bone loss without exhibiting estrogenic action in the uterus. The mechanism through which flavonoids may exert antiosteoporotic effects seems to depend, at least in part, on their mixed estrogen agonist-antagonist properties. An alternative hypothetical mechanism could derive from other biochemical actions of flavonoids such as inhibition of enzymatic activity, in particular protein kinases, or activation of an “orphan” receptor distinct from the estrogen type I receptor [31]. The results from intervention studies are still controversial. One of the potential reasons for these inconsistencies could be due to the individual differences in the flavonoids metabolism. Recently, it has been suggested that the clinical effectiveness of flavonoids might partly depend on the ability to produce equol, a gut bacterial metabolite of daidzein showing stronger estrogenic activity than the predominant flavonoids [32, 33]. 

#### 3.1.3. *Psoralea corylifolia*  L.

**Figure 3:**
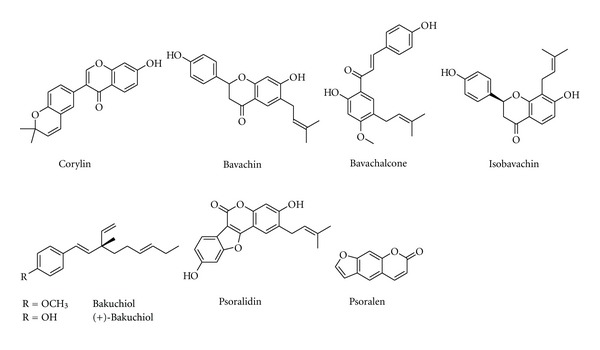
Chemical structure of compounds from *Psoralea corylifolia* L.

*Psoralea corylifolia* L. belongs to Fabaceae, the fruit is one of the commonly used herbs in formulas that are prescribed for the treatment of fractures, bone and joint diseases. Recent research suggests that *P. corylifolia* has potent oestrogenic effects and that its fruits may be a useful remedy for bone fractures, osteomalacia and osteoporosis [34]. The extract of *P. corylifolia* fruits cannot only significantly increase the concentration of inorganic phosphorus in serum, but also evidently promote bone calcification in rats. Both the extracts of its fruits and seeds and two isoflavones (corylin and bavachin, Figure 3) isolated from this plant can stimulate bone formation and have potential antiosteoporotic activity [35]. Bavachalcone (Figure 3) inhibits osteoclastogenesis by interfering with the ERK and Akt signaling pathways and the induction of c-Fos and NFATc1 during differentiation. Components derived from *P. corylifolia*, including bakuchiol, corylin, psoralidin, and isobavachin (Figure 3), have strong antioxidant activities, and corylin and bavachin have been shown to stimulate osteoblastic proliferation. Bakuchiol has a three-fold higher binding affinity for ER*α* than for ER*β*. Bakuchiol and extracts treatments had no uterotrophic activity even though they demonstrated oestrogenic activity in the in vitro assays, and reduced postmenopausal bone loss by increasing ALP, Ca concentrations, serum E_2_ concentration, and bone mineral density [36]. Psoralen (Figure 3), a coumarin-like derivative extracted from fruits of *P. corylifolia* L., has been reported to posses stimulatory effect on local new bone formation in vivo, and promote osteoblast differentiation in primary mouse calvarial osteoblasts in a dose-dependent manner by upregulation of expressions of osteoblast-specific marker genes including type I collagen, osteocalcin and bone sialoprotein and enhancement of ALP activity. It also upregulates the expression of BMP-2 and BMP-4 genes, increases the protein level of phospho-Smad1/5/8, and activates BMP reporter (12xSBE-OC-Luc) activity in a dose-dependent manner, as well as enhancing the expression of Osx, the direct target gene of BMP signaling. This suggests that psoralen acts through the activation of BMP signaling to promote osteoblast differentiation and demonstrates that psoralen could be a potential anabolic agent to treat patients with bone loss-associated diseases such as osteoporosis [37].

#### 3.1.4. *Pueraria lobata***** (Willd.) Ohwi and *P. mirifca*  Airy Shaw et Suvatabandhu

**Figure 4:**
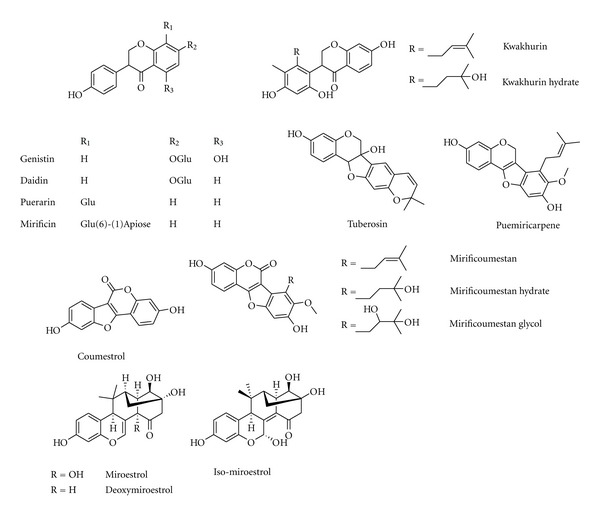
Chemical structure of compounds from *Pueraria lobata* (Willd.) Ohwi and* P. mirifca *Airy Shaw et Suvatabandhu.

*Pueraria lobata* (Willd.) Ohwi is a wild creeper plant of family Fabaceae. Its root, which is one of the earliest and most important crude herbs used in Chinese medicine for various medicinal purposes has a high content of isoflavonoids such as daidzein and genistein (Figure 2). The root of *P. lobata* shows a preventive effect on bone loss by increasing the BMD (bone mineral density) and BMC (bone mineral content) in the rats and mice of ovariectomy and orchidectomy without exhibiting estrogenic action in the uterus [38–40]. Puerarin (Figure 4), a natural isoflavonoid found in *P. lobata*, caused a significant increase in cell viability, ALP activity and mineral nodules formation in osteoblasts through activation of the PI3K/Akt pathway [41]. 

In Thailand, another species of the genus *Pueraria *plant, *P. mirifica *Airy Shaw et Suvatabandhu has been thoroughly examined for its estrogenic effects on female reproductive organs, which exhibited a higher estrogenic activity on reproductive organs than that of *P. lobata*. The long-term administration of *P. mirifica *prolongs the menstrual cycle length, suppresses folliculogenesis and ovulation in adult female monkeys, and decreases serum luteinizing hormone and follicle stimulating hormone levels, indicating that *P. mirifica *has an estrogenic effect on female reproductive systems. Phytoestrogens found in *P. mirifica *can be categorized into three groups as (i) ten isoflavonoids, comprised of daidzein, daidzin, genistin, genistein, kwakhurin, kwakhurin hydrate, tuberosin, puerarin, mirificin and puemiricarpene (Figure 4); (ii) four coumestrans, comprised of coumestrol, mirificoumestan, mirificoumestan glycol and mirificoumestan hydrate (Figure 4); and (iii) three chromenes, comprised of miroestrol, deoxymiroestrol, and isomiroestrol (Figure 4), which are rich in the plant and are known for preventing bone loss induced by estrogen deficiency [42]. *P. mirifica *dose-dependently prevents bone loss induced by orchidectomy and ovariectomy, and can be used a preventative medicine or as a therapeutic agent for the symptoms related to estrogen deficiency in menopausal women as well as in andropausal men [43].

#### 3.1.5. *Trifolium pratense*  L.

**Figure 5:**
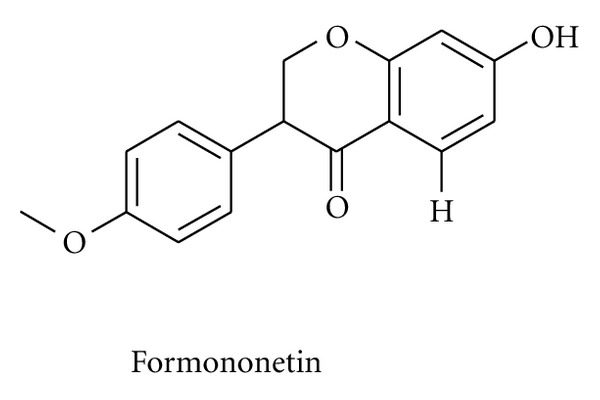
Chemical structure of formononetin.

*Trifolium pratense* (red clover) is one of the 250 species of the genus *Trifolium* belonging to Fabaceae. Red clover has been cultivated in Europe since the third or fourth century and contains four detectable estrogenic isoflavones: daidzein (Figure 2), genistein (Figure 2), formononetin (Figure 5), and biochanin A (Figure 2). Its isoflavones are effective in decreasing bone loss induced by ovariectomy, probably by reduction of the bone turnover via inhibition of bone resorption [44, 45]. Daidzein can inhibit the proliferation and differentiation of osteoclasts; this is possibly due to increasing apoptosis of osteoclast progenitors mediated by ERs. The mechanism of action of isoflavones is evidently different from that of estrogens, which have a phytoestrogen-mediated stimulation in osteoblasts rather than an inhibition in osteoclasts [46, 47].

#### 3.1.6. *Salvia miltiorrhiza*  Bunge

**Figure 6:**
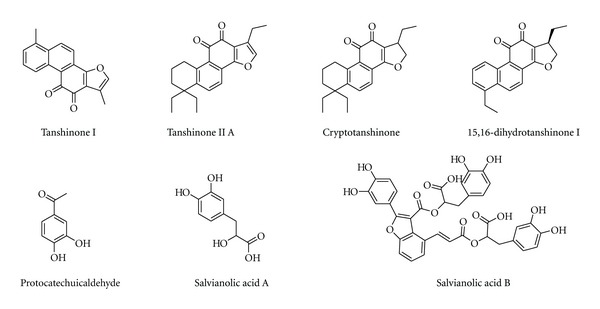
Chemical structure of compounds from *Salvia miltiorrhiza* Bunge.

*Salvia miltiorrhiza* Bunge (Labiatae), a traditional Chinese medicine, widely used in clinical practice for the prevention and treatment of cardio-cerebral vascular diseases. Pharmacological testing showed that *S. miltiorrhiza* has anticoagulant, vasodilatory, increased blood flow, anti-inflammatory, free radical scavenging, mitochondrial protective activities. Phytochemical studies revealed multiple groups of compounds from *S. miltiorrhiza *Bunge extract, the main constituents of which include tanshinones (tanshinone I, tanshinone IIA, cryptotanshinone, 15, 16-dihydrotanshinone I) and phenolics (protocatechuic aldehyde, salvianolic acid A, and salvianolic acid B) (Figure 6) [48]. *S. miltirrhiza* treatment significantly ameliorate the decrease in BMD and trabecular bone mass, decreases the TRAP activity and oxidative stress parameters including MDA (malondialdehyde) and NO (nitric oxide) induced by OVX in castrated male mice [49]. The tanshinones can reduce the formation of TRAP-positive multinuclear osteoclasts; Tanshinone IIA (Figure 6) can partially prevent ovariectomy-induced bone loss by suppressing bone turnover in vivo without stimulating osteoblast ALP activity, suppress osteoclast formation by inhibiting the expression of c-fos and NFATc1 induced by RANKL [50]. Salvianolic acid A, the aqueous bioactive component from *S. miltiorrhiza *Bunge, effectively prevents bone loss from long-term administration of prednisone in rats, protects bone from glucocorticoid induced bone marrow impairment by stimulating osteogenesis and depressing adipogenesis in bone marrow stromal cells [51]. Salvianolic acid B, another aqueous bioactive component, prevents glucocorticoid induced cancellous bone loss and decreases adipogenesis. Salvianolic acid B stimulates bone marrow stromal cell (MSC) differentiation to osteoblast and increases osteoblast activities, whilst decreasing glucocorticoid associated adipogenic differentiation through regulating the mRNA expression of PPAR-*γ*, Runx2, Dickkopf-1, and *β*-catenin in MSC [52]. 

#### 3.1.7. *Linum usitatissimum*  L.

**Figure 7:**
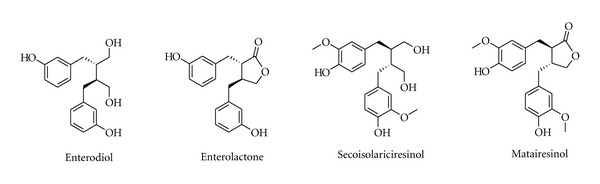
Chemical structure of compounds from *Linum usitatissimum* L.

*Linum usitatissimum* L. originally grows in Europe and warm areas of Asia, and is now widely cultivated in warm areas including America, Canada and North Europe. Its seed, also called linseed or flaxseed, can potentially exert positive effects on bone of postmenopausal women. Flaxseed is the richest source of lignans including enterodiol, enterolactone, secoisolariciresinol, and matairesinol (Figure 7), all of which are reported to have both weak estrogenic and anti-estrogenic activities [53]. Lignans are structurally similar to tamoxifen, which has beneficial effects on bone [54]. Flaxseed is also a rich source of polyunsaturated fatty acids (PUFA), especially *α*-linolenic acid. Alpha-linolenic acid may decrease the rate of bone resorption by inhibiting the biosynthesis of prostaglandins. Lignans present in flaxseed may also possess antioxidant properties. Oxygen-derived free radicals, which are formed by a number of phagocytes including monocytes, macrophages, and neutrophils, have been reported to increase chronic inflammatory diseases, aging and osteoporosis. In vivo and in vitro findings indicate that free radicals generated in the bone environment enhance osteoclast formation and bone resorption. Hence, flaxseed may reduce the rapid rate of bone loss experienced by postmenopausal women, in part, by enhancing antioxidant status [55, 56]. 

#### 3.1.8. *Drynaria fortunei*  (Kunze) J. Sm.

**Figure 8:**
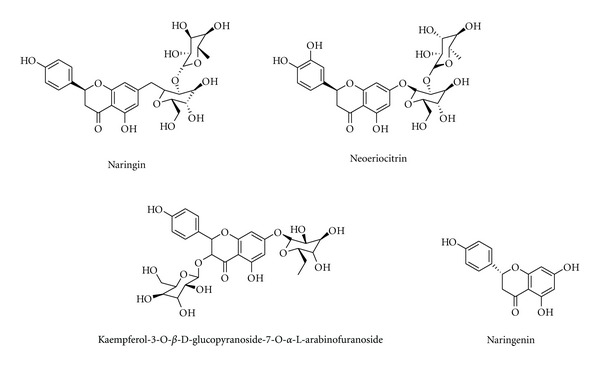
Chemical structure of compounds from *Drynaria fortunei* (Kunze) J. Sm.

The rhizome of *Drynaria fortunei* (Kunze) J. Sm., family Polypodiaceae, has a long medicinal history in the eastern Asia and is effective for the treatment of inflammation, hyperlipemia, arteriosclerosis, and gynecological diseases such as osteoporosis. The traditional Chinese and Korean prescription drugs to treat osteoporosis usually contain the rhizome of *Drynaria fortune*. In recent study, it has been found that *Drynaria fortune* has therapeutic effects on osteoporosis and bone fracture in the ovariectomized rat model, and can enhance bone formation through induction of BMP-2 and ALP, accumulation of bone matrix proteins such as type I collagen, up-regulated Runx2 and osteocalcin expression [57]. The flavonoids in *Drynaria* rhizome, including naringin, neoeriocitrin, kaempferol-3-O-*β*-D-glucopyranoside-7-O-*α*-L-arabinofuranoside (Figure 8), are antiosteoporotic chemical constituents which can activate the estrogen receptors (ERs), and replace estrogen which can be of clinical use [58]. Naringin (Figure 8) is the main active ingredients of drynariae flavonoids, which could inhibit the retinoic acid-induced osteoporosis in rats, increase BMP-2 expression and induce the bone formation, enhance the proliferation and osteogenic differentiation of human bone mesenchymal stem cells (BMSCs) in osteoporosis diseases [59, 60]. Naringin and its metabolite naringenin revealed a double directional adjusting function of estrogenic and anti-estrogenic activities primarily through selectively binding with ER, which could prevent and treat osteoporosis with the mechanism of estrogenic receptor agitation [61]. 

#### 3.1.9. *Cimicifuga racemosa* ****(L.) Nuttall

**Figure 9:**
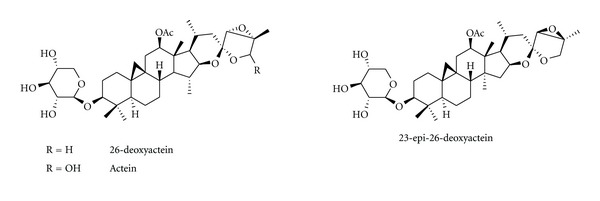
Chemical structure of compounds from *Cimicifuga racemosa *(L.) Nuttall.

*Cimicifuga racemosa* (Black cohosh), botanically a member of Ranunculaceae, has been widely used in native American therapy for a variety of ailments including dysmenorrheal and labor pains as well as for the treatment of menopausal symptoms. Black cohosh contains a number of compounds with potential bioactivity such as triterpene, glycosides, resin, salycilates, isoferulic acid, sterols, and alkaloids [62]. Black cohosh does not appear to alter the hormonal pattern associated with menopause, lower estrogen accompanied by elevated luteinizing hormone (LH), and follicle-stimulating hormone (FSH). Black cohosh results in a significant increase in trabecular bone mineral density of the proximal metaphysis of the tibia, enhances differentiation and increases the OPG-to-RANKL ratio of normal human osteoblasts [63]. Deoxyactein, including 26-deoxyactein, acetin, and 23-epi-26-deoxyactein (Figure 9), active component from black cohosh causes a significant elevation of cell growth, alkaline phosphatase activity, collagen content, and mineralization in the cells. Moreover, deoxyactein significantly decreases the production of reactive oxygen species (ROS) and osteoclast differentiation-inducing factors such as TNF-*α*, IL-6, and receptor activator of nuclear factor-*κ*B ligand in the presence of antimycin A [64, 65]. In an ovariectomized rat model of osteoporosis, extracts of black cohosh decreased urinary excretion of cross-links; however, the positive effect on trabecular BMD and on bone quality as assessed by mechanical testing was weaker than that of raloxifene. In a similar study on orchidectomized rats, extracts of black cohosh mitigated bone loss at the tibial metaphysis after 3 months. The analysis of skeletal and uterine effects by black cohosh in an ovariectomized rat model revealed weak protective effects on bone loss and on reduction of serum levels of osteocalcin and cross-laps, but no increase in uterine weight. In a small randomized controlled trial of 62 women, black cohosh alleviated menopause symptoms without affecting endometrial thickness of the uterus. However, the supplementation with black cohosh did not exhibit positive effects in severe (senile) osteopenic fracture healing as seen in early osteoporosis in rats [66]. 

#### 3.1.10. *Morinda officinalis*  How

**Figure 10:**
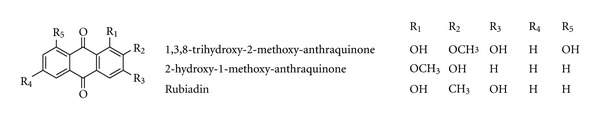
Chemical structure of compounds from *Morinda officinalis *How.

*Morinda officinalis *How belongs to family of Rubiaceae and grows in the south of China. In Chinese traditional medicine, it has been used as a kidney tonic and for strengthening bones. In a sciatic neurectomized mice model, the root extracts significantly and dose-dependently suppressed the decrease in hind limb thickness, tibia failure load, BMD, tibia Ca and P contents with an increase in serum osteocalcin levels. In addition, the root extract also significantly and dose-dependently suppressed the decrease in histomorphometric parameters of the tibia such as volume, length and thickness of trabecular bone and thickness of cortical bone in ovariectomized rats. They may act as both a suppressor of bone resorption and an enhancer of bone formation in vivo and may have some favorable effects for preventing and treating the osteoporosis induced by sciatic neurectomy and ovariectomy [67, 68]. The polysaccharides from *Morinda officinalis* can exert an increase in bone mineral density and mineral element concentration, a decrease in serum cytokines level in OVX rats [69]. The anthraquinones isolated from *M. officinalis*, have been proved to have inhibitory effects on osteoclastic bone resorption. 1,3,8-trihydroxy-2-methoxy-anthraquinone, 2-hydroxy-1-methoxy-anthraquinone and rubiadin (Figure 10) decrease the formation of bone resorption pits, the number of multinucleated osteoclasts, and the activity of tartrate resistant acid phosphates (TRAP) and cathepsin K in the coculture system of osteoblasts and bone marrow cells in the presence of 1,25-dihydroxyvitamin D3 and dexamethasone. They also enhance the apoptosis of osteoclasts induced from bone marrow cells with M-CSF and RANKL. In addition, these compounds improve the ratio of OPG and RANKL in osteoblasts, interfere with the JNK and NF-*κ*B signal pathway, and reduce the expression of calcitonin receptor (CTR) and carbonic anhydrase/II (CA II) in osteoclasts induced from bone marrow cells with M-CSF and RANKL. These findings indicate that the anthraquinone compounds from *M. officinalis* are potential inhibitors of bone resorption, and may also serve as evidence to explain the mechanism of the inhibitory effects of some other reported anthraquinones on bone loss [70, 71].

### 3.2. Antiosteoporotic Compounds Isolated from Medicinal Plants

**Figure 11:**
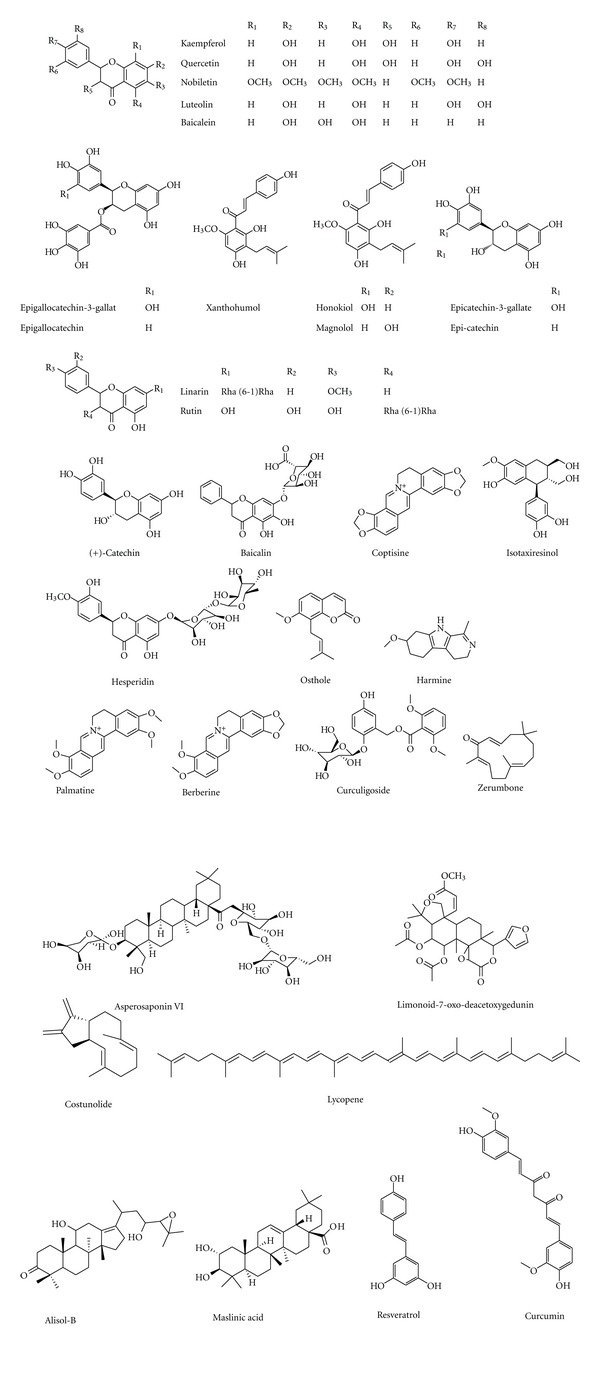
Chemical structure of compounds with antiosteoporotic activity.

A wealth of information indicates numerous bioactive components isolated from plants with antiosteoporotic potential (Table 2, Figures 1–11). These compounds can be divided into 6 categories, including flavonoids: icariin (Figure 1) [23–26], genistein (Figure 2) [29], daidzein (Figure 2) [31, 32], kaempferol (Figure 11) [72], quercetin (Figure 11) [73, 74], naringin (Figure 8) [75–77], hesperidin (Figure 11) [78], linarin (Figure 11) [79], bavachalcone (Figure 3) [36], rutin (Figure 11) [80], (+)-catechin (Figure 11) [81], nobiletin (Figure 11) [82], luteolin (Figure 11) [83], baicalein (Figure 11) [84], baicalin (Figure 1) [85], xanthohumol (Figure 11) [86]; coumarins: psoralen (Figure 3) [35], osthole (Figure 11) [87]; lignans: honokiol (Figure 11) [88, 89], isotaxiresinol (Figure 11) [90], magnolol (Figure 11) [91]; polyphenol: resveratrol (Figure 11) [92–95], curcumin (Figure 11) [96–98], tea polyphenols (including epigallocatechin-3-gallate, epigallocatechin, epi-catechin, epicatechin-3-gallate, Figure 11) [99, 100]; anthraquinones: rubiadin (Figure 10), 2-hydroxy-1-methoxy-anthraquinone (Figure 10), 1,3,8-trihydroxy-2-methoxy-anthraquinone (Figure 10) [71]; alkaloids: harmine (Figure 11) [101], coptisine (Figure 11) [102], palmatine (Figure 11) [103], berberine (Figure 11) [104, 105]; and other compounds: curculigoside (Figure 11) [106, 107], asperosaponin VI (Figure 11) [108], limonoid 7-oxo-deacetoxygedunin (Figure 11) [109], zerumbone (Figure 11) [110], costunolide (Figure 11) [111], lycopene (Figure 11) [112, 113], tanshinone IIA (Figure 6) [49, 50], salvianolic acid A (Figure 6) [51], salvianolic acid B (Figure 6) [52], alisol-B (Figure 11) [114], and maslinic acid (Figure 11) [115].

Postmenopausal bone loss appears to be associated with the estrogen deficiency that leads to excessive osteoclastic and depressed osteoblastic activity [116], and possibly also impairs intestinal absorption of calcium [117]. In recent years, evidence has been provided linking bone loss to reactive oxygen species. Estrogen deficiency induces oxidative stress, impairs bone antioxidant system in adult rats, induces increase of lipid peroxidation and H_2_O_2_, and reduction of enzymatic antioxidants like SOD (super oxygen dehydrogenises) and GSH-Px (glutathione peroxidase) in rats [118]. Some phytochemicals, which have estrogen-like and/or antioxidative activity, produce bone protective effects, via estrogen receptor and/or improving antioxidative capacity, and some may directly regulate the proliferation and activity of osteoblast and osteoclast [119]. 

Flavonoids, lignans, and coumarins, which are phyto-estrogenic constituents, modulate the bone metabolism through estrogen receptor. Icariin, genistein, daidzein, kaempferol, and costunolide have been reported to decrease bone loss through increasing osteoblast proliferation and activity, via estrogen receptor. The phytochemicals with antioxidative capacity, such as kaempferol, quercetin, linarin, naringin, resveratrol, curcumin, tea polyphenols, curculigoside, and lycopene regulate bone metabolism through reducing the production of ROS and improving antioxidative capacity. Other compounds such as bavachalcone, (+)-catechin, nobiletin, luteolin, baicalein, baicalin, harmine, berberine, honokiol, osthole and tanshinone IIA, salvianolic acid B, alisol-B, and maslinic acid and so forth directly exert effects on osteoblst and osteoclast through modulating cytokines, and regulating pathway, such as MAPK, NF-*κ*B, Wnt/*β*-catenin, and RANKL/RANK/OPG pathway.

## 4. Discussions and Conclusion

Although chemical and biochemical agents such as bisphosphonates, estrogen, and calcitonin are the mainstay in the treatment of osteoporosis and controlling fracture, they have many side effects and fail to significantly alter the course of bone fracture complications. Plants are always an exemplary source of many currently available drugs. Clinical practice and folk experience have shown the possibility of obtaining natural products to recover osteoporosis and its complications. Chinese herbs, all of which come from natural products, are thought to treat osteoporosis mainly through, tonifying kidney and improving bone quality. Numerous medicinal plants can modulate bone metabolism to reduce bone loss [120]. Therefore, biological, chemical, and pharmacological methods should be applied to screen and obtain active lead compounds from natural medicinal plants for the treatment of osteoporosis and its complications.

There are two primary types of drugs used in the treatment of osteoporosis. One is antiresorptive agents which mainly inhibit bone resorption and the other is anabolic agents which mainly build bone. Most drugs act as agents against bone resorption, such as bisphosphonates, estrogen, selective estrogen receptor modulators (SERMs), and calcitonin which could reduce bone loss, stabilize the microarchitecture of the bone, and decrease bone turnover. However, the anabolic drugs increasing bone formation are relatively rare [5]. Teriparatide, a synthetic form of parathyroid hormone, is the only anabolic agent currently approved by the US Food and Drug Administration (FDA) for the treatment of osteoporosis. The anabolic therapy is now available for those individuals who continue to fracture or lose bone on an adequate program of general prevention and antiresorptive therapy [121]. Some medicinal plants not only inhibit bone resorption, but also increase new bone formation. So these plant medicines which can increase osteoblast proliferation activity and improve bone formation should be developed to satisfy patient needs. According to the clinical needs of the patients, doctors can select antiresorptive therapy or anabolic therapy or their combination. 

There is good evidence that proper nutrition and lifestyle can promote bone health and pharmacotherapy can slow bone loss or even build new bone. However, there is still no “cure” for osteoporosis or for most other bone disorders. Those drugs that do exist, moreover, are still not ideal in terms of their expense, ease of administration, and/or side effects. When medicinal plants are being researched and developed for the treatment of osteoporosis, some questions should be considered. These questions include: (1) controllability: the effective chemical components of the drug should be clear and controllable. (2) Selectivity: the action of the drug should be specifically targeted to bone and to the molecule or rate-limiting process that is the cause of the disease. (3) Therapeutic index: the developed therapy should optimize the benefit-to-risk ratio of the drug. (4) Convenience: a more optimal drug should be the one that can be administered orally rather than parentally. 

The safety of herbal remedies should also be considered. Although the popular view that herbals are natural and harmless, some herbal toxic effects have also be reported out of which, the hepatotoxicity is the most frequently reported toxic effect [122]. The investigation of compounds and composite formula regarding safety and toxicity is needed before definitive clinical guidelines can be made. On the other hand, the medicinal plants lack standardization; this makes it difficult to validate the plant use, and may discourage further studies. However, the chances of finding an active compound in a plant traced from ethnobotanical information are significantly higher than random chance in conventional techniques. Plants which are utilized often should be investigated for pharmacological and therapeutic effects in patients suffering from osteoporosis.

It is obvious that many plants have the potential to prevent and treat osteoporosis however, only a fraction of these plants have been thoroughly investigated so far. More efficient and reliable bioassays should be developed as a matter of urgency to systematically evaluate the antiosteoporotic efficacy of plant extracts, to identify the bioactive compounds responsible for the bone protective manifestation, and to elucidate antiosteoporotic mechanisms. In addition, as most antiosteoporotic agents from medicinal plants are prophylactic in nature rather than therapeutic and clinical trials have not yet been undertaken, the application of herbal agents are restricted. If such studies are encouraged and performed more herbal drugs for human use may soon be available.

## Figures and Tables

**Table 1:** Antiosteoporotic medicinal plants.

Family	Scientific name	Plant parts used	Reported relevant ethnomedical uses	Pharmacological study/chemical constituents	Reference
Amaranthaceae	*Achyranthes bidentata* Blume	Root	Bone related diseases	Decrease bone loss in OVX rats by inhibiting osteoclast formation/oleanolic acid glycosides, ecdysone and allantoin	[123, 124]
Amaryllidaceae	*Curculigo orchioides *Gaertn.	Rhizome	Impotence, tinnitus	Decrease bone loss by inhibiting bone resorption/phenolic glycosides	[106, 125]
Apiaceae	*Cnidium monnieri* (L.) Cuss.	Fruit	Impotence, lumbar pain	Reverse prednisone-induced bone mass loss, inhibit the high bone turnover; enhance osteoblastic proliferation and differentiation, inhibit formation and maturation of osteoclast/coumarins	[87, 126, 127]
Apiaceae	*Cuminum cyminum *L*. *	Fruit	Toothache, diarrhea, epilepsy	Prevent ovariectomy—induced bone loss/*β*-sitosterol, stigmasterol, luteolin and apigenin	[128]
Apiaceae	*Ferula hermonis *Boiss	Root	Frigidity, impotence	Prevent bone loss caused by severe estrogen deficiency by regulating calcium mobilization and mitochondrial permeability/daucane sesquiterpenes, ferutinin	[129]
Apiaceae	*Angelica sinensis* (Oliv.) Diels	Root	Hematopoietic, abnormal or painful menstruation, other women's diseases	Increase ALP activity and synthesis of collagenase type I of osteoblast/ligustilide, butylidene phihalide, ferulic acid	[130]
Araliaceae	*Panax notoginseng* (Burk.) F. H. Chen	Root	Trauma, injury of muscles, bone fracture	Prevent bone loss and deterioration of trabecular microarchitecture, stimulate proliferation and differentiation of osteoblast/triterpene saponins	[131, 132]
Araliaceae	*Acanthopanax senticosus* (Rupr. et Maxim.) Harms	Stem	Hypertension, rheumatism, ischemic heart disease, diabetes	Decrease bone loss in postmenopausal women/acanthosides, eleutherosides, senticoside, triterpen saponin, flavones	[133]
Berberidaceae	*Epimedium brevicornu* Maxim	Leaf	Impotence, prospermia, hyperdiuresis, osteoporosis, menopause syndrome, rheumatic arthritis, hypertension and chronic tracheitis		[16–26]
Berberidaceae	*Epimedium koreanum *Nakai	Leaf		See section 3.1.1	
Berberidaceae	*Epimedium pubescens *Maxim	Leaf			
Berberidaceae	*Epimedium sagittatum *(Sieb. et Zucc.)	Leaf			
Berberidaceae	*Berberis aristata* DC	Stem bark	Menopausal disorders, osteoporosis	Decrease bone loss/berberine chloride, palmatine chloride, magnoflavine, canadine, berberastine, obaberine, columbavine and talifendine	[134, 135]
Brassicaceae	*Lepidium meyenii *Walp.	Root	Hot flushes, tender breast, vaginal dryness, osteoporosis	Improve the bone mass in OVX rats/macaridine, macaene, macamides, and maca alkaloids	[136, 137]
Campanulaceae	*Platycodon grandiflorum* (Jacq.) A. DC.	Root	Cough, chronic diseases	Stimulate osteoblast differentiation through p38 MAPK and ERK signaling pathways/saponin	[138]
Caprifoliaceae	*Sambucus williamsii* Hance	Stem and ramulus	Inflammation, bone fractures, joint diseases	Suppress the OVX-induced increase in bone turnover, inhibit bone resorption, stimulate bone formation/lignans	[139]
Compositae	*Carthamus tinctorius *L.	Seed	Ankyloenteron, rheumatism, and chronic nephritis	Prevent bone loss through modulation ALP and IGF-1/lignans, flavones, serotonins	[140, 141]
Compositae	*Silybum marianun *(L.) Gaertn	Seed	Liver disease	Prevent bone loss in rats induced by OVX with mild proliferative effects in uterus/silibinin, isosilibinin, silydianin and silychristin	[142, 143]
Compositae	*Wedelia calendulacea *Less.	Flower	Liver disorders, jaundice, uterine hemorrhage, menorrhagia	Promote bone formation, decrease bone loss/isoflavones and wedelolactone	[144]
Compositae	*Artemisia iwayomogi* Kitamura	Aerial parts	Diabetes and hepatitis	Stimulate bone formation/phenolic compounds	[145]
Convolvulaceae	*Cuscuta chinensis* Lam.	Seed	Sexual dysfunction, osteoporosis, senescence	Enhance osteoblast differentiation and mineralization/quercetin, kaempferol, isorhamnetin, hyperoside and astragalin	[146, 147]
Davalliaceae	*Davallia formosana* Hayata	Rhizome	Bone disease, osteoporosis	Prevent bone loss, enhance bone strength, inhibit the deterioration of trabecular microarchitecture via inhibition of bone resorption/(−)-epicatechin 3-O-*β*-D-allopyranoside	[148]
Dicksoniaceae	*Cibotium barometz* (L.) J. Sm.	Rhizome	Lumbago, rheumatism, polyuria, leucorrhoea	Prevent bone loss induced by ovariectomy, inhibit osteoclast formation	[149]
Dioscoreaceae	*Dioscorea alata* L.	Rhizome	Dyspnea, spermatorrhea, leucorrhagia, diabetes	Increase bone formation by inducing mesenchymal stem cells differentiation into osteoblasts	[150]
Dioscoreaceae	*Dioscorea spongiosa *J. Q. Xi et al.	Rhizome	Rheumatoid arthritis, bone disorder	Inhibit the decrease in bone mineral density, stimulate proliferation and mineralization of osteoblast, inhibit formation and bone resorption of osteoclast/seroidal saponins	[151, 152]
Dipsacaceae	*Dipsacus asperoides* C. Y. Cheng et T. M. Ai	Root	Traumatic ecchymoma, injury of muscles, bone fractures	Inhibit bone loss induced by ovariectomy, enhance osteoblast maturation and differentiation by increasing BMP-2 synthesis and activating p38 and ERK1/2/asperosaponin VI	[108, 153]
Ericaceae	*Vaccinium angustifolium *Aiton	Fruit	Cardiovascular disease	Prevent bone loss in ovarian hormone deficiency, stimulate osteoblast differentiation and reduce mesenchymal stromal cell senescence/phenolic acids (gallic acid, p-hydroxybenzoic acid, chlorogenic, p-coumaric, caffeic, ferulic and ellagic acids), flavonoids (anthocyanins, catechin, epichatechin, quercetin, kaempferol and myrecetin)	[154]
Eucommiaceae	*Eucommia ulmoides *Oliv.	Bark	Hypertension, renal injury	Prevent estrogen deficiency-induced bone loss, increase osteoblast proliferation and inhibit differentiation of osteoclast/lignans, iridoids, flavonoids and terpenoids	[155–157]
Euphorbiaceae	*Emblica officinalis *Gaertn.	Fruit	Dyslipidemia, atherosclerosis	Induce osteoclast apoptosis through downregulating the expression of IL-6 and NF-*κ*B	[158]
Fabaceae	*Erythrina variegata *Linn	Stem bark	Stomachache, rheumatism, eye ailments, swellings	Suppress the bone loss by inhibiting osteoclast differentiation and maturation/genistein derivatives	[159, 160]
Fabaceae	*Glycine max *(Linn.) Merr.	Seed	Cardiovascular disease, cancer, osteoporosis, renal function	See section 3.1.2	[27–33]
Fabaceae	*Onobrychis ebenoides* Boiss. et Spruner	Whole plant	Estrogenic activity	Decrease bone loss without affecting body and uterine weight/isoflavones (ebenosin, afrormosin, formononetin and daidzein), benzofurans and benzoypyrans (ebenfuran I, ebenfuran II and ebenfuran III )	[161–163]
Fabaceae	*Psoralea corylifolia *L*. *	Fruit	Bone fracture, osteomalacia and osteoporosis	See section 3.1.3	[34–37]
Fabaceae	*Pueraria lobate *(Willd.) Ohwi	Root	Influenza, hypertension, angina pectoris	See section 3.1.4	[38–41]
Fabaceae	*Pueraria mirifica *Airy Shaw et Suvatabandhu	Root	Reproductive organs, cardiovascular diseases, climacteric related symptoms	See section 3.1.4	[42, 43]
Fabaceae	*Rhynchosia volubilis* Lour.	Seed	Toothache, rheumatic arthritis, snake bite	Facilitate osteoblastic MG-63 cell proliferation/genistein and daidzein	[164]
Fabaceae	*Sophora japonica *L.	Fruit	Hematochezia, bleeding hemorrhoids	Suppress formation and differentiation of osteoclast/isoflavonoids	[165, 166]
Fabaceae	*Butea monosperma *(L.) Kuntze	Stem bark	Bone fracture	Prevent OVX-induced bone loss by stimulating bone formation/methoxyisoflavones (cajanin, isoformononetin, cladrin and medicarpin)	[167]
Fabaceae	*Phaseolus vulgaris* L	Seed	Estrogenic activity	Prevent estrogen deficiency-induced osteopenia without affecting the uterine mass	[168]
Fabaceae	*Trifolium pratense* L.	Aerial parts	Menopause symptoms, cardiovascular disease	See section 3.1.5	[44–47]
Ginkgoaceae	*Ginkgo biloba* Linn.	Leaf	Cardiovascular disease	Reverse bone loss in glucocorticoid-induced osteoporosis and mandibular osteoporosis/kaempferol, quercetin, isorhamnetin, and terpenoids (ginkgolides and bilobalides)	[169, 170]
Juglandaceae	*Juglans regia* L.	Fuit	Heart disease, prostate cancer, hyperlipidemic	Induce nodule formation of osteoblast/ellagic acid, *α*-tocopherol, fatty acids, flavonoids and phenolic acids	[171]
Labiatae	*Ajuga decumbens *Thunb.	Whole plant	Hypertension, hemoptysis, carbuncles and joint pain	Downregulate the differentiation of osteoclast, upregulate mineralization of osteoblast-like MC3T3-E1 cells	[172]
Labiatae	*Salvia miltiorrhiza *Bge	Root	Cardiovascular diseases	See section 3.1.6	[48–52]
Lauraceae	*Cinnamomum cassia *(L.) C. Presl	Bark	Dyspepsia, gastritis, blood circulation disturbances, inflammatory diseases	Stimulate bone formation in vitro and may contribute to the prevention of osteoporosis and inflammatory bone diseases/cinnamic aldehyde, cinnamic alcohol, cinnamic acid, and coumarin	[173]
Liliaceae	*Allium cepa* L.	Bulb	Insomnia, hyperglycemic, Hyperlipidemic	Decrease the ovariectomy-induced bone resorption via attenuation of RANKL—induced ERK, p38, and NF-*κ*B activation	[174]
Liliaceae	*Allium sativum* L.	Bulb	Influenza, dysentery, tuberculosis	Prevent bone loss, reverse the low BMD and low tensile strength caused by ovariectomy/allicin, allylmethyltrisulphide, diallyldisulphide, ajoene, monoterpenes (citral, geraniol and linalool), and flavonoids (quercetin and rutin)	[175]
Liliaceae	*Anemarrhena asphodeloides* Bge.	Rhizome	Lung disease, fever, diabetes and constipation	Prevent OVX-induced bone loss in rats through the promotion of bone formation but not the inhibition of bone resorption/steroidal saponins	[176]
Liliaceae	*Polygonatum sibiricum* Red.	Rhizome	Hypotension, Hyperglycemic, Hyperlipidemic	Prevent bone loss/polysaccharide	[177]
Linaceae	*Linum usitatissimum *L.	Seed	Postmenopausal osteoporosis	See section 3.1.7	[53–56]
Lythraceae	*Heimia myrtifolia* Cham.	Leaf	Osteoporosis	Stimulate formation and mineralization of osteoblastic cell lines HOS58 and saos-2/vertine (cryogenine), lythrine, lythridine, polyphenols	[178]
Malvaceae	*Abelmoschus manihot *(L.) Medik.	Leaf	Chronic glomerulonephritis	Reduce bone loss in conditions of estrogen deficiency/calcium	[179]
Menispermaceae	*Tinospora cordifolia* (Willd.) Miers	Stem	Dyspepsia, fever, urinary diseases	Estrogenic activity, prevent bone loss in ovariectomized rats/alkaloids, terpenoids, glycosides, sterols, lactones and fatty acids	[180]
Myrsinaceae	*Labisia pumila* var. *alata *(Scheff.) Mez.	Root	Menstrual irregularities, painful menstruation	Prevent the changes in bone biochemical markers but failed to prevent the bone calcium loss induced by ovariectomy/C15 monoene resorcinols, phenolic compounds, flavonoids	[181]
Oleaceae	*Ligustrum lucidum* Ait.	Fruit	Menopausal problems, tinnitus, rheumatic pains, palpitations, insomnia symptoms	Improve bone properties in aged rats via increasing osteoblast formation and mineralization/oleanolic acid, ursolic acid, acetyloleanolic acid	[182, 183]
Orchidaceae	*Anoectochilus formosanus* Hayata	Whole plants	Lung disease, pleurodynia, abdominal pain, fever, hypertension and snake bites	Suppress the bone loss caused by estrogen deficiency through suppression of RANKL expression required for osteoclast formation.	[184]
Orobanchaceae	*Cistanche deserticola* Y. C. Ma	Stem	Forgetfulness, loss of hearing, chronic constipation.	Enhanced bone mineral density and bone mineral content/harmine	[101]
Orobanchaceae	*Cistanche salsa* (C. A. Mey.) G. Beck	Stem	Kidney deficiency, neurasthenia	Suppress bone loss in ovariectomized mice/(2E, 6R)-8-hydroxy-2, 6-dimethyl-2-octenoic acid	[185]
Pleurotaceae	*Pleurotus eryngii *(De Candolle: Fr.) Quel.	Fruiting body	Liver, kidney and gastrointestinal disorders	Alleviate the decrease in the trabecular bond mineral density in ovariectomized rats, increase the ALP activity and secretion of osteoprotegerin, improve the osteocalcin mRNA and Runx2 gene expression in osteoblasts; Decrease the number of tartrate-resistant acid phosphatase (TRAP)-positive multinucleated cells and resorption areas of osteoclast	[186]
Polypodiaceae	*Drynaria fortunei* (Kunze) J. Sm.	Rhizome	Bone fractures and joint diseases	See section 3.1.8	[57–61]
Punicaceae	*Punica granatum* Linn.	Fruit	Parasitic infections, ulcers, diarrhea, dysentery, hemorrhage, respiratory pathologies	Increase bone volume and trabecular number, and decrease trabecular separation in OVX rats/genistein, daidzein, ellagitannins and ellagic acid	[187]
Ranunculaceae	*Cimicifuga foetida *L.	Rhizome	Cooling and detoxification agent	Inhibit osteoclastic bone resorption, increase BMD in OVX mice/oxidized cycloartane-type triterpenoids and phenol type derivatives	[188]
Ranunculaceae	*Cimicifuga racemosa *(L.) Nuttall	Rhizome	Dysmenorrhea, labor pains, menopausal symptoms	See section 3.1.9	[62–66]
Rosaceae	*Prunus mume* Sieb et ZUCC.	Fruit	Chronic gastritis	Increase alkaline phosphatase activity, cell proliferation and mineralization, enhance the expression of BMP-2 of osteoblast/citric acid, malic acid, chlorogenic acid and 5-hydroxymethy-furfural	[189, 190]
Rosaceae	*Rubus coreanus* Miq.	Fruit	Impotence, spermatorrhoea, and back pain	Prevent bone loss caused by estrogen deficiency by dual regulation of the enhancement of osteoblast function and induction of osteoclast apoptosis/ellagic acid, fupenzic acid, *β*-sitosterol	[191, 192]
Rubiaceae	*Morinda officinalis* How	Root	Rheumatism	See Section 3.1.10	[67–71]
Rutaceae	*Poncirus trifoliata* (L.) Raf.	Fruit	Gastritis, dysentery, digestive tract ulcers, uterine contraction, and cardiovascular diseases	Inhibit glucocorticoid-induced bone loss by decreasing expression of anxA6/flavone (poncirin, hesperidin, rhoifolin, naringin, neohesperidin)	[193]
Rutaceae	*Citrus paradisi* Macf.	Fruit	Digestion system, lose weight	Improve bone quality by enhancing bone mineral deposition in ORX rats/vitamin C, hesperidin and limonoids	[194, 195]
Scrophulariaceae	*Rehmannia glutinosa* Libosch	Root	Haemostatic, cardiotonic, and diuretic agent	Increase ALP activity and the expression of the OPG of osteoblast, decrease the number of TRAP-positive MNCs and the resorption areas of osteoclast, alleviate the decrease in the trabecular BMD, and increase the cortical bone thickness ovariectomy-induced osteoporotic rats/luteolin, mannitol, stigmasterol, campesterol, catalpol, rehmannin.	[196]
Solanaceae	*Withania somnifera *Dunn.	Root	Nerve diseases and anxiety	Inhibit bone loss in ovariectomized rats/withanolides	[197]
Taxaceae	*Taxus yunnanensis* cheng et L.K.	Seed, bark	Cancer	Increase bone mineral content and bone mineral density in ovariectomized rats/isotaxiresinol, taxol, harringtonine	[90]
Theaceae	*Stewartia koreana* Nakai ex Rehd.	Leaf	Inflammatory diseases	Inhibit osteoclast differentiation and prevent inflammatory bone loss/spinasterol glycoside	[198]
Ulmaceae	*Ulmus davidiana *Planch.	Bark	Oedema, mastitis, gastric cancer and inflammation	Promote osteoblastic differentiation by increasing bone morphogenic protein-2 as well as ALP mRNA expression in MC3T3-E1 cells, inhibit bone resorption/davidianones A, B, and C, mansonones E, F, H, and I	[199]
Ulmaceae	*Ulmus wallichiana* Planch.	Bark	Bone fracture	Mitigate ovariectomy-induced osteoporosis in rats, stimulate osteoblast function and inhibit osteoclast differentiation/quercetin-6-C-*β*-D-glucopyranoside	[200–203]
Verbenaceae	*Vitex agnus-castus* L.	Fruit	Premenstrual symptoms, climacteric complaints	Protect bone in orchidectomized rats/apigenin, cascitin, and dopaminergic compounds	[204]
Vitaceae	*Cissus quadrangularis* L.	Aerial parts, root	Hemorrhoids, menstrual disorders, scurvy, flatulence, bone fractures, bone diseases	Prevent bone loss in ovariectomized rats, stimulate osteoblastogenesis through up-regulation of MAPK-dependent alkaline phosphatase activity/*β*-sitosterol, *δ*-amyrin, *δ*-amyrone, favanoids (quercetin), 6′-O-trans-cinnamoyl-catalpol	[205–207]
Zingiberaceae	*Curcuma comosa* Roxb.	Rhizome	Postpartum, uterine bleeding, inflammation	Prevent bone loss induced by estrogen deficiency/diarylheptanoids, curcumin	[208, 209]

**Table 2:** Antiosteoporotic compounds isolated from medicinal plants.

Compound	Pharmacological activity	reference
*Flavonoids *		
Icariin	See Section 3.1.1	[23–26]
Genistein	See Section 3.1.2	[29]
Daidzein	prevent bone loss in ovariectomized rats and orchidectomized rats; inhibit osteoclastic differentiation and bone resorption by increasing the activity of mature osteoblasts via ER*β*, regulating RUNX 2/Cbf*α*1 production, and stimulating the secretion osteoprotegerin.	[31, 32]
Kaempferol	increase ALP activity in cultured human MG-63 osteoblasts through ERK and ER pathway; prevent antimycin A-induced cell damage in mitochondrial membrane potential dissipation, complex IV inactivation, ROS production through activation of PI3K (phosphoinositide 3-kinase), Akt (protein kinase B), CREB (cAMP-response element-binding protein) in MC3T3-E1.	[72]
Quercetin	reverse the decreased biomechanical quality and the impaired microarchitecture of the femurs in diabetic rats through improving antioxidant capacity; inhibit osteoclastic differentiation and bone resorption via inducing apoptosis and involving NF-*κ*B and AP-1.	[73, 74]
Naringin	protect against retinoic acid-induced osteoporosis and improve bone quality in rats; perturb osteoclast formation and bone resorption by inhibiting RANK-mediated NF-*κ*B and ERK signaling; induce bone morphogenetic protein-2 expression via PI3K, Akt, c-Fos/c-Jun and AP-1 pathway in osteoblasts; prevent hydrogen peroxide-induced dysfunction in osteoblastic MC3T3-E1 cells.	[75–77]
Hesperidin	protect bone loss in OVX rats, improve BMD and femoral load in intact rats	[78]
Linarin	protect osteoblasts against hydrogen peroxide-induced osteoblastic dysfunction, exert antiresorptive actions via the reduction of RANKL and oxidative damage	[79]
Bavachalcone	inhibit osteoclastogenesis by interfering with the ERK and Akt signaling pathways and the induction of c-Fos and NFATc1.	[36]
Rutin	inhibit ovariectomy—induced trabecular bone loss in rats by slowing down resorption and increasing osteoblastic activity.	[80]
(+)-Catechin	enhance cell survival, alkaline phosphatase activity, decrease bone-resorbing cytokines (TNF-*α* and IL-6) production and apoptosis in osteoblasts.	[81]
Nobiletin	prevent bone loss in ovariectomized rats; suppress formation and bone resorption of osteoclast induced by interleukin-1; suppress the expression of cyclooxygenase-2, NF-*κ*B-dependent transcription, and prostaglandin E production in osteoblasts.	[82]
Luteolin	increase bone mineral density and bone mineral content of trabecular and cortical bones in the femur of OVX rats; inhibit the differentiation of both bone marrow mononuclear cells and RAW 264.7 cells into osteoclasts and the bone resorptive activity of osteoclasts.	[83]
Baicalein	inhibit the differentiation and bone resorptive activity of osteoclasts by inhibiting RANKL-induced activation of signaling molecules (Akt, ERK/MAP kinase and NF-*κ*B) and mRNA expression of osteoclast-associated genes TRAP, matrix metalloproteinase 9 and c-Src, c-Fos, Fra-2 and NFATc1.	[84]
Baicalin	promote osteoblastic differentiation via Wnt/*β*-catenin signaling and enhance the mRNA expression of osteoprotegerin	[85]
Xanthohumol	upregulate ALP activity and expression of osteogenic marker genes by activation of RUNX2 via mechanisms related to the p38 MAPK and ERK signaling pathway	[86]
*Coumarins *		
Psoralen	promote osteoblast differentiation by up-regulation of expressions of osteoblast-specific marker through the activation of BMP signaling	[35]
Osthole	prevent bone loss and improve bone microarchitecture, histomorphometric parameters, and biomechanical properties in OVX rats; stimulate osteoblast proliferation and differentiation through *β*-catenin/BMP signaling.	[87]
*Lignan *		
Honokiol	increase cell growth, alkaline phosphatase activity, collagen synthesis, mineralization, glutathione content, and osteoprotegerin release in the osteoblast; decrease the production of TNF-*α*, IL-6, and RANKL in the presence of antimycin A; stimulate osteoblastogenesis by suppressing NF-*κ*B activation.	[88, 89]
Isotaxiresinol	improve bone mineral content, bone mineral density, and bone strength indexes in OVX control rats; slightly increase bone formation and significantly inhibit bone resorption	[90]
Magnolol	cause a significant elevation of cell growth, alkaline phosphatase activity, collagen synthesis, mineralization, and glutathione content in osteoblast; decrease the production of osteoclast differentiation inducing factors such as RANKL, TNF-*α*, and IL-6 in the presence of antimycin A	[91]
*Polyphenol *		
Resveratrol	prevent osteoporosis induced by cyclosporin A; inhibit the differentiation and bone resorbing activity of osteoclasts through inhibition of ROS production; promote the formation of osteoblasts by induction of bone morphogenetic protein-2 through Src kinase-dependent estrogen receptor activation; promote osteogenesis of human mesenchymal stem cells by upregulating RUNX2 gene expression via the SIRT1/FOXO3A axis.	[92–95]
Curcumin	improve bone microarchitecture and mineral density in APP/PS1 transgenic mice; improve bone strength and biochemical marker in ovariectomized mature rat model; inhibit OVX-induced bone loss by reducing osteoclastogenesis through increasing antioxidant activity and impairing RANKL signaling.	[96–98]
Tea polyphenols (including epigallocatechin-3-gallate, epigallocatechin epi-catechin epicatechin-3-gallate)	attenuate trabecular and cortical bone loss through increasing bone formation while suppressing bone resorption due to its antioxidant capacity; inhibit the formation and differentiation of osteoclasts via inhibition of matrix metalloproteinases.	[99, 100]
*Anthraquinones *		
Rubiadin; 2-hydroxy-1-methoxy-anthraquinone; 1,3,8-trihydroxy-2-methoxy-anthraquinone	decrease bone resorption, the number of multinucleated osteoclasts, and the activity TRAP and cathepsin K of osteoclast; induce the apoptosis of osteoclasts through improving the ratio of OPG and RANKL in osteoblasts, interfering with the JNK and NF-*κ*B signal pathway, and reducing the expression of calcitonin receptor and carbonic anhydrase/II in osteoclasts.	[71]
*Alkaloids *		
Harmine	prevent bone loss in ovariectomized osteoporosis model mice; inhibit osteoclast formation and bone resorption via downregulation of c-Fos and NFATc1 induced by RANKL.	[101]
Coptisine	inhibit RANKL-induced NF-*κ*B phosphorylation in osteoclast precursors; suppress the formation, differentiation and bone resorption of osteoclast through regulation of RANKL and OPG gene expression in osteoblastic cells	[102]
Palmatine	inhibit osteoclast formation and bone resorption in the co-culture system with mouse bone marrow cells (BMC) and osteoblasts; induce disruption of actin ring formation in mature osteoclasts with an impact on cell viability	[103]
Berberine	prevent bone loss in SAMP6 senile osteoporosis model and ovariectomized rats; inhibit formation and differentiation of osteoclast; promote osteoblast differentiation through activation of Runx2 by p38 MAPK.	[104, 105]
*Other compounds *		
Curculigoside	inhibit bone loss in ovariectomized mice; promote the proliferation and differentiation of osteoblast; prevent hydrogen peroxide-induced dysfunction and oxidative damage in calvarial osteoblasts; inhibit the formation, differentiation and bone resorption of osteoclast.	[106, 107]
Asperosaponin VI	induce osteoblast maturation and differentiation, and bone formation via increasing BMP-2 synthesis and activating p38 and ERK1/2 pathway	[108]
Limonoid 7-oxo-deacetoxygedunin	inhibit RANKL-induced osteoclastogenesis by suppressing activation of the NF-*κ*B and MAPK pathways	[109]
Zerumbone	abolish RANKL-induced NF-*κ*B activation, inhibit osteoclastogenesis, and suppress human breast cancer—induced bone loss in athymic nude mice	[110]
Costunolide	stimulate the growth and differentiation of osteoblastic MC3T3-E1 cells, which may be associated with ER, PI3K, PKC, and MAPK signaling pathway	[111]
Lycopene	reduce oxidative stress and the levels of bone turnover markers in postmenopausal women; stimulate proliferation and alkaline phosphatase activity of osteoblasts; inhibit osteoclasts formation and bone resorption activity.	[112, 113]
Tanshinone IIA	inhibit osteoclast differentiation and bone resorption through disruption of the actin ring by inhibiting c-Fos and NFATc1 expression.	[49, 50]
Salvianic acid A	prevent bone loss from long-term administration of prednisone in rats; protect bone from glucocorticoid—induced bone marrow impairment by stimulating osteogenesis and depressing adipogenesis in bone marrow stromal cells.	[51]
Salvianolic acid B	prevent glucocorticoid—induced cancellous bone loss and decrease adipogenesis; stimulate bone marrow stromal cell differentiation to osteoblast and increase osteoblast activities; decrease glucocorticoid—induced associated adipogenic differentiation through regulating the mRNA expression of PPAR-*γ*, Runx2, Dickkopf-1 and *β*-catenin in MSC	[52]
Alisol-B	prevent bone loss in mice; inhibit osteoclastogenesis by inhibiting the phosphorylation of JNK, and expression of NFATc1 and c-Fos; suppresses 2-methylene-19-nor-(20S)-1*α*, 25(OH)_2_D_3_—induced hypercalcemia as resulting from the inhibition of osteoclastogenesis	[114]
Maslinic acid	suppress osteoclastogenesis and prevent ovariectomy-induced bone loss by regulating RANKL-mediated NF-*κ*B and MAPK signaling pathway	[115]
